# Supplementary material for: Dynamic gain and frequency comb formation in exceptional-point lasers
Source: Nat Commun. 2024 Oct 4;15:8618. doi: 10.1038/s41467-024-52957-4 (PMC11452692; doi:10.1038/s41467-024-52957-4)
Supplement: Supplementary file 1 — Supplementary Information [file 41467_2024_52957_MOESM1_ESM.pdf]

Supplementary Information for Dynamic gain and frequency comb formation in exceptional-point lasers

CONTENTS

1. Maxwell–Bloch equations in 3D 1
2. Single-mode lasing solution and active-cavity resonance operator 2
3. Dynamic inversion factor ζ 4
4. Single-mode stability analysis and the comb threshold 5
5. PALT for direct-bandgap semiconductor lasers 7
6. System parameters and properties 9
7. PALT solution through volume integral equation 9
8. FDTD simulations of the Maxwell–Bloch equations 11
9. Exact-EP laser 12

References 15

1. MAXWELL–BLOCH EQUATIONS IN 3D

In this section, we derive the 3D Maxwell–Bloch (MB) equations, namely Eqs. (1)–(3) of the main text, from the evolution equation of the atomic density matrix. Here, the active part of the gain medium is modeled by an ensemble of two-level atoms. For each atom, let $\hat{H}_0$ be the atomic Hamiltonian in the absence of external electric fields, with $|a\rangle$ and $|b\rangle$ being the ground state and excited state respectively, at energies $\hbar\omega_a$ and $\hbar\omega_b$. In the presence of an external electrical field $\tilde{\mathbf{E}}$, the total Hamiltonian $\hat{H}$ includes the electric dipole energy $e\hat{\mathbf{r}} \cdot \tilde{\mathbf{E}}$,

$$\hat{H} = \hat{H}_0 + e\tilde{\mathbf{E}} \cdot \hat{\mathbf{r}}, \tag{S1}$$

where $\hat{\mathbf{r}}$ is the position operator of the two-level atom, with $\langle a|\hat{\mathbf{r}}|a\rangle = \langle b|\hat{\mathbf{r}}|b\rangle = \mathbf{0}$ given its spatial symmetry.

The ensemble of atoms can be described by a density matrix $\hat{\rho}$, which satisfies

$$\frac{\partial \hat{\rho}}{\partial t} = \frac{1}{i\hbar}[\hat{H}, \hat{\rho}] + \text{incoherent terms}, \tag{S2}$$

with the brackets denoting a commutator. The "incoherent terms" here include pumping from the ground state to the excited state at rate γ_{ab}, spontaneous emission from the excited state to the ground state at rate γ_{ba}, and dephasing of the off-diagonal elements of the density matrix at rate $\gamma_\perp$. In the basis of $|a\rangle$ and $|b\rangle$,

$$\begin{bmatrix} \rho_{aa} & \rho_{ab} \\ \rho_{ba} & \rho_{bb} \end{bmatrix} = \begin{bmatrix} \langle a|\hat{\rho}|a\rangle & \langle a|\hat{\rho}|b\rangle \\ \langle b|\hat{\rho}|a\rangle & \langle b|\hat{\rho}|b\rangle \end{bmatrix}, \tag{S3}$$

the elements of the density matrix then evolve as

$$\frac{\partial}{\partial t}\rho_{aa} = \gamma_{ba}\rho_{bb} - \gamma_{ab}\rho_{aa} - \frac{1}{i\hbar}\tilde{\mathbf{E}} \cdot (\rho_{ba}\mathbf{R}^* - \text{c.c.}), \tag{S4}$$

$$\frac{\partial}{\partial t}\rho_{bb} = \gamma_{ab}\rho_{aa} - \gamma_{ba}\rho_{bb} + \frac{1}{i\hbar}\tilde{\mathbf{E}} \cdot (\rho_{ba}\mathbf{R}^* - \text{c.c.}), \tag{S5}$$

$$\frac{\partial}{\partial t}\rho_{ba} = -(i\omega_{ba} + \gamma_\perp)\rho_{ba} + \frac{1}{i\hbar}(\rho_{bb} - \rho_{aa})\tilde{\mathbf{E}} \cdot \mathbf{R}, \tag{S6}$$

where $\mathbf{R} = -e\langle b|\hat{\mathbf{r}}|a\rangle$ is the atomic dipole moment, $\omega_{ba} = \omega_b - \omega_a$ is the frequency gap, and c.c. denotes complex conjugate. Let N be the number density of the two-level atoms, so that $N_a = N\rho_{aa}$ and $N_b = N\rho_{bb}$ are the population

densities at the ground level and the excited level, respectively. The expectation of the gain-induced polarization is then $\tilde{\mathbf{P}} = -eN\text{tr}(\hat{\rho}\hat{\mathbf{r}}) = \mathbf{P} + \mathbf{P}^*$, where $\mathbf{P} = N\rho_{ba}\mathbf{R}^*$. Multiplying Eqs. (S4)–(S5) with N and Eq. (S6) with $N\mathbf{R}^*$, we get

$$\frac{\partial}{\partial t}N_a = \gamma_{ba}N_b - \gamma_{ab}N_a - \frac{1}{i\hbar}\tilde{\mathbf{E}}\cdot(\mathbf{P}-\mathbf{P}^*), \tag{S7}$$

$$\frac{\partial}{\partial t}N_b = \gamma_{ab}N_a - \gamma_{ba}N_b + \frac{1}{i\hbar}\tilde{\mathbf{E}}\cdot(\mathbf{P}-\mathbf{P}^*), \tag{S8}$$

$$\frac{\partial}{\partial t}\mathbf{P} = -(i\omega_{ba}+\gamma_\perp)\mathbf{P} + \frac{1}{i\hbar}D(\tilde{\mathbf{E}}\cdot\mathbf{R})\mathbf{R}^*, \tag{S9}$$

where $D = N_b - N_a$ is the population inversion. Subtracting Eq. (S7) from Eq. (S8) yields

$$\frac{\partial}{\partial t}D = -\gamma_\parallel(D - D_\text{p}) + \frac{2}{i\hbar}\tilde{\mathbf{E}}\cdot(\mathbf{P}-\mathbf{P}^*), \tag{S10}$$

where $\gamma_\parallel = \gamma_{ab}+\gamma_{ba}$ is the decay rate of the inversion, and $D_\text{p} = N(\gamma_{ab}-\gamma_{ba})/(\gamma_{ab}+\gamma_{ba})$ is the net pumping strength. Eqs. (S9)–(S10) together with Maxwell's equations constitute the MB equations in 3D.

For the analytical and numerical analysis, it is more convenient to work with MB equations with dimensionless units. Let $R = \sqrt{\mathbf{R}\cdot\mathbf{R}^*}$ be the amplitude and $\theta = \mathbf{R}/R$ be the unit vector of the atomic dipole moment. We normalize D and D_p in the units of $R^2/(\varepsilon_0\hbar\gamma_\perp)$, $\tilde{\mathbf{E}}$ in the units of $2R/(\hbar\sqrt{\gamma_\perp\gamma_\parallel})$, and $\mathbf{P}$ in the units of $2R/(\varepsilon_0\hbar\sqrt{\gamma_\perp\gamma_\parallel})$. Then the dimensionless MB equations read

$$\frac{\partial}{\partial t}D = -\gamma_\parallel(D - D_\text{p}) - \frac{i\gamma_\parallel}{2}\tilde{\mathbf{E}}\cdot(\mathbf{P}-\mathbf{P}^*), \tag{S11}$$

$$\frac{\partial}{\partial t}\mathbf{P} = -(i\omega_{ba}+\gamma_\perp)\mathbf{P} - i\gamma_\perp D(\tilde{\mathbf{E}}\cdot\theta)\theta^*, \tag{S12}$$

$$\nabla\times\tilde{\mathbf{B}} = \frac{1}{c^2}\left(\varepsilon_c\frac{\partial\tilde{\mathbf{E}}}{\partial t} + \frac{\sigma}{\varepsilon_0}\tilde{\mathbf{E}} + \frac{\partial\tilde{\mathbf{P}}}{\partial t}\right), \tag{S13}$$

$$\nabla\times\tilde{\mathbf{E}} = -\frac{\partial\tilde{\mathbf{B}}}{\partial t}. \tag{S14}$$

Our implementation of FDTD simulations (Sec. 8 below) is based directly on Eqs. (S11)–(S14).

We can write the real-valued field $\tilde{\mathbf{E}}$ as $\tilde{\mathbf{E}}(\mathbf{r},t) = \mathbf{E}(\mathbf{r},t)+\mathbf{E}^*(\mathbf{r},t)$. The spectrum of the lasing field $\mathbf{E}(\mathbf{r},t)$ is centered near the peak-gain frequency of ω_{ba} with a bandwidth no greater than the gain bandwidth $\gamma_\perp \ll \omega_{ba}$. Meanwhile, the spectrum of $\mathbf{E}^*(\mathbf{r},t)$ is centered near $-\omega_{ba}$. Similarly for $\mathbf{P}$ and $\mathbf{P}^*$. Therefore, the $\mathbf{E}\cdot\mathbf{P}$ term and the $\mathbf{E}^*\cdot\mathbf{P}^*$ term in Eq. (S11) will oscillate approximately as $e^{-2i\omega_{ba}t}$ and $e^{2i\omega_{ba}t}$ respectively. Since $\omega_{ba}\sim 10^{15}$ rad/s is six orders of magnitude greater than $\gamma_\parallel \sim 10^9$ s^{-1} for semiconductor lasers operating at optical frequencies, these oscillations average away before the gain medium can respond, and we can drop the $\mathbf{E}\cdot\mathbf{P}$ and $\mathbf{E}^*\cdot\mathbf{P}^*$ terms in Eq. (S11). Similarly the $\mathbf{E}^*$ term in the right-hand side of Eq. (S12) will oscillate near $-\omega_{ba}$ while the gain-induced polarization $\mathbf{P}$ oscillates near ω_{ba}, so we can also drop the $\mathbf{E}^*$ term in the right-hand side of Eq. (S12). With this rotating-wave approximation, Eqs. (S11)–(S14) become

$$\frac{\partial}{\partial t}D = -\gamma_\parallel(D - D_\text{p}) - \frac{i\gamma_\parallel}{2}(\mathbf{E}^*\cdot\mathbf{P} - \mathbf{E}\cdot\mathbf{P}^*), \tag{S15}$$

$$\frac{\partial}{\partial t}\mathbf{P} = -(i\omega_{ba}+\gamma_\perp)\mathbf{P} - i\gamma_\perp D(\mathbf{E}\cdot\theta)\theta^*, \tag{S16}$$

$$-\nabla\times\nabla\times\mathbf{E} - \frac{1}{c^2}\left(\varepsilon_c\frac{\partial^2}{\partial t^2} + \frac{\sigma}{\varepsilon_0}\frac{\partial}{\partial t}\right)\mathbf{E} = \frac{1}{c^2}\frac{\partial^2}{\partial t^2}\mathbf{P}, \tag{S17}$$

These Eq. (S15)–(S17) are the MB equations, Eqs. (1)–(3) of the main text, that we analyze below.

2. SINGLE-MODE LASING SOLUTION AND ACTIVE-CAVITY RESONANCE OPERATOR

In the subsequent sections, we will build our analysis upon the steady-state solution in the single-mode lasing regime, which features a real-valued lasing frequency ω_0 with[1,2]

$$D(\mathbf{r},t) = D_0(\mathbf{r}), \tag{S18}$$

$$\mathbf{P}(\mathbf{r},t) = P_0(\mathbf{r})\theta^* e^{-i\omega_0 t}, \tag{S19}$$

$$\mathbf{E}(\mathbf{r},t) = \mathbf{E}_0(\mathbf{r})e^{-i\omega_0 t}. \tag{S20}$$

We can verify that Eqs. (S18)–(S20) constitute an exact solution of the MB equations, Eqs. (S15)–(S17), when

$$D_0(\mathbf{r}) = \frac{D_\text{p}(\mathbf{r})}{1+|\Gamma_0 E_0(\mathbf{r})|^2}, \tag{S21}$$

$$P_0(\mathbf{r}) = \Gamma_0 D_0(\mathbf{r}) E_0(\mathbf{r}), \tag{S22}$$

$$\left[-\nabla\times\nabla\times + \frac{\omega_0^2}{c^2}\left(\varepsilon_c(\mathbf{r}) + \frac{i\sigma(\mathbf{r})}{\omega_0\varepsilon_0} + \Gamma_0 D_0(\mathbf{r})\theta^*\theta\cdot\right)\right]\mathbf{E}_0(\mathbf{r}) = 0, \tag{S23}$$

where $\Gamma_0 = \Gamma(\omega_0)$, $E_0 \equiv \mathbf{E}_0\cdot\theta$, and

$$\Gamma(\omega) \equiv \frac{\gamma_\perp}{\omega - \omega_{ba} + i\gamma_\perp}. \tag{S24}$$

Eq. (S23) is repeated as Eq. (4) in the main text.

To facilitate discussions in the next two sections, we introduce the active-cavity wave operator in this single-mode regime

$$\hat{O}(\omega) = -\nabla\times\nabla\times + \frac{\omega^2}{c^2}\varepsilon_\text{eff}(\mathbf{r},\omega), \tag{S25}$$

with an outgoing boundary condition, where the effective permittivity profile is

$$\varepsilon_\text{eff}(\mathbf{r},\omega) = \varepsilon_c(\mathbf{r}) + \tilde{\varepsilon}(\mathbf{r},\omega), \qquad \tilde{\varepsilon}(\mathbf{r},\omega) = \frac{i\sigma(\mathbf{r})}{\omega\varepsilon_0} + \Gamma(\omega) D_0(\mathbf{r})\theta^*\theta\,\cdot\,. \tag{S26}$$

We separate out the gain and loss contributions in $\tilde{\varepsilon}(\mathbf{r},\omega)$, which is generally much smaller than the passive contribution $\varepsilon_c(\mathbf{r})$. Then, Eq. (S23) can be written as

$$\hat{O}(\omega_0)\mathbf{E}_0 = 0. \tag{S27}$$

Eq. (S27) is nonlinear in $\mathbf{E}_0(\mathbf{r})$ because the saturated gain $D_0(\mathbf{r})$ in $\tilde{\varepsilon}(\mathbf{r},\omega)$ of the operator $\hat{O}(\omega)$ depends on $\mathbf{E}_0(\mathbf{r})$. For a given pump strength, after obtaining ω_0 and $\mathbf{E}_0(\mathbf{r})$ by solving Eq. (S27) as a nonlinear equation, it is convenient to consider operator $\hat{O}(\omega)$ with a $D_0(\mathbf{r})$ fixed (frozen) by that pre-computed $\mathbf{E}_0(\mathbf{r})$ but with a variable ω. This "active cavity wave operator" $\hat{O}(\omega)$ defines the resonances[3] $\{\psi_n(\mathbf{r})\}$ (namely, eigenmodes with an outgoing boundary condition) of the active cavity at that pump strength,

$$\hat{O}(\tilde{\omega}_n)\psi_n = 0, \tag{S28}$$

where $\tilde{\omega}_n$ is the complex-valued eigen frequency of resonance ψ_n. Since the gain in operator $\hat{O}$ is fixed, Eq. (S28) is linear in ψ_n. By construction, the lasing mode $\mathbf{E}_0$ is a resonance of the active cavity with a real-valued eigenvalue of $\tilde{\omega}_n = \omega_0$.

When the pumping strength is below the first lasing threshold D_1^th, the steady-state solution is $\mathbf{E}(\mathbf{r},t) = 0$, which trivially satisfies the MB equations, Eqs. (S15)–(S17). Below the first threshold D_1^th, we can consider operator $\hat{O}$ with an unsaturated gain (since $\mathbf{E} = 0$), and track its set of eigenvalues $\{\tilde{\omega}_n\}$. All of the eigenvalues should have a negative imaginary part, corresponding to an exponential decay in time. The first threshold is reached when one of the eigenvalues $\tilde{\omega}_n$ reaches the real-frequency axis, corresponding to a cavity resonance having enough gain to overcome its radiation loss and absorption loss. With pumping at and above D_1^th, that eigenvalue stays on the real-frequency axis as ω_0, and the corresponding resonance becomes the lasing mode $\mathbf{E}_0$. The amplitude of the lasing mode is determined self-consistently by solving the nonlinear Eq. (S27).

In the "steady-state *ab initio* laser theory" (SALT),[1,2] the same recipe is continued above the first lasing threshold D_1^th. One would track the eigenvalues of the resonances of the active cavity given $\mathbf{E}_0$ at each pump strength. The next threshold D_2^th is reached when the eigenvalue of another resonance (excluding $\mathbf{E}_0$) of the active cavity reaches the real-frequency axis. We will show in Sec. 4 that such a recipe is rigorous in the limit of $\gamma_\parallel \to 0$, where the gain medium is static. When $\gamma_\parallel$ is not negligible, the dynamics of the gain medium can modify the stability of the single-mode solution such that the next threshold is no longer simply a resonance of the active cavity reaching the real-frequency axis.

3. DYNAMIC INVERSION FACTOR ζ

In Sec. II of the main text, we consider a perturbation to the single-mode lasing solution, Eqs. (S18)–(S23) above. Here, we derive the dynamic inversion factor ζ that quantifies the degree of frequency coupling, producing $\mathbf{E}_{-1}e^{-i\omega_{-1}t}$ from $\mathbf{E}_1 e^{-i\omega_1 t}$ in the perturbation. Our starting point is Eq. (6) of the main text, rewritten below as

$$\begin{aligned}\hat{O}(\omega_{-1})\mathbf{E}_{-1} &= -\frac{\omega_{-1}^2}{c^2}\frac{\Gamma_{-1}(\Gamma_0-\Gamma_1^*)}{2}\frac{\gamma_\parallel}{i\gamma_\parallel-\omega_d}D_0E_0^2E_1^*\theta^* \\ &\approx \frac{\omega_{-1}^2}{c^2}\frac{\gamma_\parallel}{i\gamma_\parallel-\omega_d}D_0E_0^2E_1^*\theta^*,\end{aligned} \tag{S29}$$

where $E_m \equiv \mathbf{E}_m\cdot\theta$, $\Gamma_m = \Gamma(\omega_m)$, $\omega_m = \omega_0 + m\omega_d$. We consider $|\omega_m - \omega_{ba}| \ll \gamma_\perp$, so that $\Gamma_m \approx -i$.

To solve Eq. (S29), we expand $\mathbf{E}_{-1}$ in the non-orthogonal basis of the active-cavity resonances $\{\psi_n\}$ in Eq. (S28),

$$\mathbf{E}_{-1} = \sum_n \alpha_n\psi_n. \tag{S30}$$

Substituting Eq. (S30) into Eq. (S29), subtracting Eq. (S28), and approximating $\varepsilon_{\text{eff}}(\mathbf{r},\omega)\approx\varepsilon_c(\mathbf{r})$, we obtain

$$\sum_n \alpha_n(\omega_{-1}^2-\tilde{\omega}_n^2)\varepsilon_c\psi_n \approx \omega_{-1}^2\frac{\gamma_\parallel}{i\gamma_\parallel-\omega_d}D_0E_0^2E_1^*\theta^*. \tag{S31}$$

To proceed, we want to solve Eq. (S31) for the expansion coefficients $\{\alpha_n\}$. The set of resonances $\{\psi_n\}$ are not orthogonal but are biorthogonal,[3] which we can utilize to project out α_n in Eq. (S31). Consider the Green's vector identity,[4]

$$\psi_m\cdot(\nabla\times\nabla\times\psi_n)-\psi_n\cdot(\nabla\times\nabla\times\psi_m) = \nabla\cdot[\psi_n\times(\nabla\times\psi_m)-\psi_m\times(\nabla\times\psi_n)]. \tag{S32}$$

Using the approximation $\varepsilon_{\text{eff}}(\mathbf{r},\omega)\approx\varepsilon_c(\mathbf{r})$ again, from Eq. (S28), we get $\nabla\times\nabla\times\psi_n \approx \frac{\tilde{\omega}_n^2}{c^2}\varepsilon_c\psi_n$, so

$$\psi_m\cdot(\nabla\times\nabla\times\psi_n)-\psi_n\cdot(\nabla\times\nabla\times\psi_m) \approx \frac{\tilde{\omega}_n^2-\tilde{\omega}_m^2}{c^2}\varepsilon_c\psi_m\cdot\psi_n \tag{S33}$$

Substituting Eq. (S33) into Eq. (S32), taking the volume integration over the laser cavity, and applying the divergence theorem, we get

$$\frac{\tilde{\omega}_n^2-\tilde{\omega}_m^2}{c^2}\int_V \varepsilon_c\psi_m\cdot\psi_n dr^3 \approx \oint\!\!\!\oint_{\mathbf{S}}[\psi_{\mathbf{n}}\times(\nabla\times\psi_{\mathbf{m}})-\psi_{\mathbf{m}}\times(\nabla\times\psi_{\mathbf{n}})]\cdot d\mathbf{s}, \tag{S34}$$

where V is the volume of the laser cavity and $\mathbf{S}$ is its surface. To proceed, we assume that the resonances $\{\psi_n\}$ of interest have high-enough quality factors that the radiation field on the cavity surface is much weaker than the interior field, and the right-hand side of Eq. (S34) is negligible compared to the left-hand-side. Under such approximation, we obtain the biorthogonality relationship,

$$\int_V \varepsilon_c\psi_m\cdot\psi_n dr^3 \approx 0 \text{ when } \tilde{\omega}_m \neq \tilde{\omega}_n. \tag{S35}$$

Multiplying Eq. S31 with ψ_n, integrating over V, and using the biorthogonality relationship Eq. (S35), we obtain the coefficient α_n of interest,

$$\alpha_n \approx \frac{\omega_{-1}^2}{\omega_{-1}^2-\tilde{\omega}_n^2}\frac{\gamma_\parallel}{i\gamma_\parallel-\omega_d}\frac{\langle D_0E_0^2E_1^*\psi_n\cdot\theta^*\rangle}{\langle\varepsilon_c\psi_n\cdot\psi_n\rangle}, \tag{S36}$$

where $\langle\cdots\rangle = \int_V\cdots dr^3$ denotes integration over V. We can see that the coefficient α_n is enhanced when (1) the resonance eigen frequency $\tilde{\omega}_n$ is close to ω_{-1}, (2) the frequency detuning $\omega_d = \omega_1-\omega_0$ between the perturbation and the lasing frequency is small, (3) the spatial overlap $|\langle D_0E_0^2E_1^*\psi_n\cdot\theta^*\rangle|^2$ between the excitation source profile and the resonance profile is large, and (4) the Petermann factor $K_n \equiv |\langle\varepsilon_c|\psi_{\mathbf{n}}|^2\rangle/\langle\varepsilon_c\psi_{\mathbf{n}}\cdot\psi_{\mathbf{n}}\rangle|^2$ of the resonance is large.

Among the set $\{\alpha_n\}$, typically one coefficient will be much larger than the other coefficients. We denote the largest coefficient as α_ψ, with ψ denoting the corresponding resonance and ω_ψ its complex-valued eigen frequency. Such a ω_ψ is typically close to ω_{-1}, so

$$\alpha_\psi \approx \frac{\omega_{-1}}{2(\omega_{-1}-\omega_\psi)}\frac{\gamma_\parallel}{i\gamma_\parallel-\omega_d}\frac{\langle D_0E_0^2E_1^*\psi\cdot\theta^*\rangle}{\langle\varepsilon_c\psi\cdot\psi\rangle}. \tag{S37}$$

Keeping only the dominant contribution, $\mathbf{E}_{-1} \approx \alpha_\psi \psi$, we obtain the stationary-inversion factor ζ,

$$\frac{\langle|\mathbf{E}_{-1}|^2\rangle}{\langle|\mathbf{E}_1|^2\rangle} \approx |\alpha_\psi|^2 \frac{\langle|\psi|^2\rangle}{\langle|\mathbf{E}_1|^2\rangle} \approx \frac{\gamma_\parallel^2}{\omega_d^2+\gamma_\parallel^2} \frac{\omega_{-1}^2/4}{|\omega_{-1}-\omega_\psi|^2} \frac{\langle|\psi|^2\rangle}{\langle|\mathbf{E}_1|^2\rangle} \left| \frac{\langle D_0 E_0^2 E_1^* \psi \cdot \theta^*\rangle}{\langle \varepsilon_c \psi \cdot \psi\rangle} \right|^2 \equiv \zeta. \tag{S38}$$

Taking $\psi = \mathbf{E}_0$ to be the resonance closest to ω_{-1}, approximating $\omega_{-1}^2 \approx \omega_0^2$, and taking θ to be real-valued, we obtain Eq. (7) of the main text.

When ω_{-1} is near a degeneracy, there may be two resonances that both contribute significant. If it is a Hermitian degeneracy due to symmetry, we can take ψ to be the superposition of the two near-degenerate modes that maximizes the spatial overlap $|\langle D_0 E_0^2 E_1^* \psi_n \cdot \theta^*\rangle|^2$ with the excitation source profile. If it is a non-Hermitian degeneracy, the two resonances will have similar spatial profiles, so the ζ factor will be comparable for the two resonances; using either one can give an order-of-magnitude estimate of $\langle|\mathbf{E}_{-1}|^2\rangle/\langle|\mathbf{E}_1|^2\rangle$. Because of the non-orthogonality of the basis $\{\psi_n\}$, projecting onto only one resonance ψ is not quantitatively accurate. For example, approaching an EP, the Petermann factor (and the associated projection onto one resonance of the EP pair) can diverge, even though the total projection onto both resonances remains finite due to a cancellation of divergences.[5] However, the single-resonance projection can already provide an order-of-magnitude estimate. For the system considered in Figs. 2–3 of the main text, we obtain $\zeta \approx 0.26$ from Eq. (S38), reasonably close to the actual $\langle|\mathbf{E}_{-1}|^2\rangle/\langle|\mathbf{E}_1|^2\rangle \approx 0.10$ computed from PALT just above the comb threshold $D_2^{\mathrm{th}} = D_{\mathrm{c}}^{\mathrm{th}}$, even though the Petermann factor $K_n = 28$ is already very large.

4. SINGLE-MODE STABILITY ANALYSIS AND THE COMB THRESHOLD

Here, we perform a general stability analysis on the single-mode steady-state lasing solution, Eqs. (S18)–(S23) above. As the pumping strength is increased above the first threshold D_1^{th}, the next threshold D_2^{th} will be reached when the single-mode solution becomes unstable. This second threshold D_2^{th} is typically when a second mode turns on. For a laser sufficiently close to an EP that the ζ factor is not negligible, this threshold $D_2^{\mathrm{th}} = D_{\mathrm{c}}^{\mathrm{th}}$ is where a frequency comb emerges. In any of these scenarios, the stability can be determined by perturbing Eqs. (S18)–(S23) with a small perturbation,[6]

$$D(\mathbf{r},t) = D_0(\mathbf{r}) + \delta D(\mathbf{r},t), \tag{S39}$$
$$\mathbf{P}(\mathbf{r},t) = [P_0(\mathbf{r}) + \delta P(\mathbf{r},t)]\theta^* e^{-i\omega_0 t}, \tag{S40}$$
$$\mathbf{E}(\mathbf{r},t) = [\mathbf{E}_0(\mathbf{r}) + \delta \mathbf{E}(\mathbf{r},t)] e^{-i\omega_0 t}, \tag{S41}$$

and analyzing whether the perturbation decays or grows in time. Plugging Eqs. (S39)–(S41) into Eqs. (S15)–(S17) and keeping only terms linear to the perturbation (since the perturbation is small), we get

$$\frac{\partial}{\partial t}\delta D = -\gamma_\parallel \delta D - \frac{i\gamma_\parallel}{2}(\delta E^* P_0 + E_0^* \delta P - \delta E P_0^* - E_0 \delta P^*), \tag{S42}$$
$$\frac{\partial}{\partial t}\delta P = i(\omega_0 - \omega_{ba} + i\gamma_\perp)\delta P - i\gamma_\perp(\delta D E_0 + D_0 \delta E), \tag{S43}$$
$$-\nabla\times\nabla\times\delta\mathbf{E} - \frac{1}{c^2}\left[\varepsilon_c \left(\frac{\partial}{\partial t} - i\omega_0\right)^2 + \frac{\sigma}{\varepsilon_0}\left(\frac{\partial}{\partial t} - i\omega_0\right)\right]\delta\mathbf{E} = \frac{1}{c^2}\left(\frac{\partial}{\partial t} - i\omega_0\right)^2 \delta P \theta^*, \tag{S44}$$

where $\delta E = \delta\mathbf{E}\cdot\theta$. The zeroth-order terms reduce to Eq. (S21)–(S23) and are always satisfied. Eqs. (S42)–(S44) apply to any perturbation. Since they define a linear dynamical system for $\delta\mathbf{E}(\mathbf{r},t)$, $\delta\mathbf{E}^*(\mathbf{r},t)$, $\delta P(\mathbf{r},t)$, $\delta P^*(\mathbf{r},t)$, and $\delta D(\mathbf{r},t)$, the time evolution of any perturbation can be written as a superposition of the eigenvectors of the linear system. Therefore, for the purpose of a stability analysis, it suffices to characterize the dynamics of the eigenvectors (namely, the eigenvalues).

In previous work,[6–9] such an eigen analysis was carried out by separating the real and imaginary parts of Eqs. (S42)–(S44) and considering an aggregated real-valued vector $u = (\mathrm{Re}\,\delta\mathbf{E}, \mathrm{Im}\,\delta\mathbf{E}, \mathrm{Re}\,\delta\mathbf{P}, \mathrm{Im}\,\delta\mathbf{P}, \delta D)$. The resulting equations are suitable for numerical solution but have changed so much from the original wave equations that it is difficult to analyze them, interpret the spectrum and other physical quantities, or relate them to the single-resonance-turn-on picture of SALT.

In the preceding derivation of the dynamic inversion factor $\zeta \approx |\mathbf{E}_{-1}|^2/|\mathbf{E}_1|^2$, we showed that a monochromatic perturbation $\mathbf{E}_1 e^{-i(\omega_0+\omega_{\mathrm{d}})t}$ necessarily results in another frequency component $\mathbf{E}_{-1}e^{-i(\omega_0-\omega_{\mathrm{d}})t}$ when ζ is not negligible. We also showed that $\mathbf{E}_{\pm1}$, $\mathbf{P}_{\pm1}$, and $D_{\pm1}$ are all proportional to the perturbation. Also, from the $\delta E^* P_0$ term in Eq. (S42), we see that a perturbation with $e^{-i\omega_d t}$ time dependence [note that $e^{-i\omega_0 t}$ is already factored out from the

perturbation in Eqs. (S40)–(S41)] will generate $e^{i\omega_d^* t}$ time dependence. Therefore, here we consider a multi-spectral perturbation with both ω_d and $-\omega_d^*$ frequency components as the trial eigenvector,

$$\delta D(\mathbf{r},t) = D_1(\mathbf{r})e^{-i\omega_d t} + D_1^*(\mathbf{r})e^{i\omega_d^* t}, \tag{S45}$$

$$\delta P(\mathbf{r},t) = P_1(\mathbf{r})e^{-i\omega_d t} + P_{-1}(\mathbf{r})e^{i\omega_d^* t}, \tag{S46}$$

$$\delta \mathbf{E}(\mathbf{r},t) = \mathbf{E}_1(\mathbf{r})e^{-i\omega_d t} + \mathbf{E}_{-1}(\mathbf{r})e^{i\omega_d^* t}. \tag{S47}$$

The "frequency difference" ω_d here is the eigenvalue of the linear perturbation problem and is complex-valued in general. Substituting into Eqs. (S42)–(S44), we can verify that the ansatz of Eqs. (S45)–(S47) has the correct time dependencies and constitutes a valid eigenvector for the linear dynamical system.

From the $e^{-i\omega_d t}$ and $e^{i\omega_d^* t}$ terms of Eq. (S42)–(S44), we obtain

$$D_1 = 0.5\Gamma_\parallel(E_{-1}^* P_0 + E_0^* P_1 - E_1 P_0^* - E_0 P_{-1}^*), \tag{S48}$$

$$P_{\pm 1} = \Gamma_\pm (D_{\pm 1} E_0 + D_0 E_{\pm 1}), \tag{S49}$$

$$-\nabla\times\nabla\times\mathbf{E}_{\pm 1} + \frac{\omega_{\pm 1}}{c^2}\left(\varepsilon_c + \frac{i\sigma}{\omega_{\pm 1}\varepsilon_0}\right)\mathbf{E}_{\pm 1} = -\frac{\omega_{\pm 1}}{c^2}P_{\pm 1}\theta^*, \tag{S50}$$

where $\omega_1 = \omega_0 + \omega_d$, $\omega_{-1} = \omega_0 - \omega_d^*$, $\Gamma_\parallel \equiv \gamma_\parallel/(\omega_d + i\gamma_\parallel)$, $\Gamma_\pm = \Gamma(\omega_{\pm 1})$, and $D_{-1} = D_1^*$. Substituting Eqs. (S22) and (S49) into Eq. (S48), we can eliminate the gain-induced polarization to yield

$$D_1 = 0.5\Gamma_\parallel\left[(\Gamma_+ - \Gamma_-^*)|E_0|^2 D_1 + (\Gamma_+ - \Gamma_0^*)D_0 E_0^* E_1 + (\Gamma_0 - \Gamma_-^*)D_0 E_0 E_{-1}^*\right], \tag{S51}$$

from which D_1 can be solved as

$$D_1 = D_0(\chi_+ E_1 + \chi_- E_{-1}^*), \tag{S52}$$

with

$$\chi_+ = \frac{0.5\Gamma_\parallel(\Gamma_+ - \Gamma_0^*)E_0^*}{1 - 0.5\Gamma_\parallel(\Gamma_+ - \Gamma_-^*)|E_0|^2}, \tag{S53}$$

$$\chi_- = \frac{0.5\Gamma_\parallel(\Gamma_0 - \Gamma_-^*)E_0}{1 - 0.5\Gamma_\parallel(\Gamma_+ - \Gamma_-^*)|E_0|^2}. \tag{S54}$$

Substituting Eqs. (S49) and (S52) into Eq. (S50), we can further eliminate D_1 and obtain an eigenvalue problem for $\mathbf{E}_{\pm 1}(\mathbf{r})$ along:

$$\hat{O}(\omega_1)\mathbf{E}_1 + \frac{\omega_1^2}{c^2}\Gamma_+ D_0 E_0(\chi_+ E_1 + \chi_- E_{-1}^*)\theta^* = 0, \tag{S55}$$

$$\hat{O}(\omega_{-1})\mathbf{E}_{-1} + \frac{\omega_{-1}^2}{c^2}\Gamma_- D_0 E_0(\chi_+^* E_1^* + \chi_-^* E_{-1})\theta^* = 0, \tag{S56}$$

where $\hat{O}(\omega)$ is the active-cavity wave operator defined in Eq. (S25).

Eqs. (S55)–(S56) show that the two spectral components $\mathbf{E}_{\pm 1}(\mathbf{r})e^{-i\omega_{\pm 1}t}$ of the perturbation are explicitly coupled through $E_0\chi_\pm$, which arises from the dynamic inversion $D_1 e^{-i\omega_d t}$ and is proportional to its relaxation rate $\gamma_\parallel$. We can take the complex conjugate of Eq. (S56), so that Eq. (S55) and [Eq. (S56)]* together forms a coupled eigenproblem for $\mathbf{E}_1$ (with an outgoing boundary condition) and $\mathbf{E}_{-1}^*$ (with an incoming boundary condition). Solving this coupled eigenproblem yields the complex eigenvalue ω_d and the associated perturbation in Eqs. (S45)–(S47). As a reminder, $\omega_{\pm 1}$, $\Gamma_\pm$, $\chi_\pm$, and the $\Gamma_\parallel$ in $\chi_\pm$ all depend on ω_d, so this is a nonlinear eigenvalue problem.

Eqs. (S55)–(S56) always have a trivial solution with $\omega_d = 0$ and $(\mathbf{E}_1, \mathbf{E}_{-1}) = (A, -A^*)\mathbf{E}_0$, where $A = A_r + iA_i$ is any constant, such that the perturbation $\delta\mathbf{E}(\mathbf{r},t)e^{-i\omega_0 t} = 2iA_i\mathbf{E}_0(\mathbf{r})e^{-i\omega_0 t}$ is marginally stable and is ± 90-degree out-of-phase with the lasing mode $\mathbf{E}_0(\mathbf{r})e^{-i\omega_0 t}$. Here, the coupling terms are cancelled, $\chi_+ E_1 + \chi_- E_{-1}^* = 0$, and Eqs. (S55)–(S56) are satisfied since $\hat{O}(\omega_0)\mathbf{E}_0 = 0$. This trivial solution must exist because there is a gauge degree of freedom in the single-mode solution Eq. (S20): $\mathbf{E}(\mathbf{r},t) = e^{i\phi}\mathbf{E}_0(\mathbf{r})e^{-i\omega_0 t}$ with any real-valued global phase ϕ is also a solution of the MB equations.

At the first threshold D_1^{th}, $\mathbf{E}_0 = 0$, so the coupling strength $E_0\chi_\pm$ is zero, and Eqs. (S55)–(S56) decouple to $\hat{O}(\omega_{\pm 1})\mathbf{E}_{\pm 1} = 0$, same as Eq. (S28). Therefore, the set of stability eigenvalues $\{\omega_d\}$ above D_1^{th} is continuously connected to $\{\tilde{\omega}_n - \omega_0\}$ below D_1^{th}, where $\{\tilde{\omega}_n\}$ is the set of eigen frequencies of the active-cavity resonances with

an unsaturated gain, defined in Eq. (S28). Above the first threshold $D_1^{\rm th}$, $\mathbf{E}_0 \neq 0$, so Eqs. (S55)–(S56) are no longer equivalent to Eq. (S28), and each stability eigenmode evolves into a superposition of the cavity resonances.

Above $D_1^{\rm th}$, we can track how the set of complex eigenvalues $\{\omega_d\}$ evolves with the pumping strength. Near $D_1^{\rm th}$, all eigenvalues (except the trivial one, $\omega_{\rm d} = 0$) have a negative imaginary part, so any perturbation $\delta\mathbf{E}(\mathbf{r},t)$, $\delta P(\mathbf{r},t)$, and $\delta D(\mathbf{r},t)$ has to either decay exponentially in time or merely change the global phase of the lasing mode; here, the single-mode lasing solution Eqs. (S18)–(S23) is stable. When one of the non-trivial ω_d reaches the real-frequency axis, single-mode lasing is no longer stable since any perturbation that overlaps with the eigenvector with that real-valued ω_d will start to grow exponentially. Therefore, the crossing of ω_d with the real-frequency axis marks the second threshold $D_2^{\rm th}$. Just above that threshold, the perturbations $\mathbf{E}_1$ and $\mathbf{E}_{-1}$ continuously evolve into two comb lines in Eq. (11) of the main text while that ω_d stays on the real axis and becomes the comb spacing.

A stationary-inversion system corresponds to the limit of $\gamma_\parallel \to 0$, where $\chi_\pm \to 0$, the dynamic inversion D_1 in the perturbation eigenvector vanishes, and $\mathbf{E}_{\pm 1}$ in Eqs. (S55)–(S56) decouple. Each of the decoupled $\mathbf{E}_{\pm 1}$ follows $\hat{O}(\omega_{\pm 1})\mathbf{E}_{\pm 1} = 0$, same as Eq. (S28). In this limit, the stability eigenmodes are the resonances $\{\psi_n\}$ of the active cavity $\varepsilon_{\rm eff}$. This reduces to the formalism of SALT,[1,2] where the second threshold $D_2^{\rm th}$ corresponds to a second individual resonance of the active cavity receiving enough gain to overcome its loss and reach the real-frequency axis.

For a dynamic system where $\gamma_\parallel$ is not negligible—more specifically, when the dynamic inversion factor $\zeta \approx |\mathbf{E}_{-1}|^2/|\mathbf{E}_1|^2$ is not negligible—the eigenmode $(\mathbf{E}_1 e^{-i\omega_1 t}, \mathbf{E}_{-1} e^{-i\omega_{-1} t})$ in Eqs. (S55)–(S56) is a multi-spectral perturbation that comes with a dynamic inversion D_1. In this case, $\mathbf{E}_1$ (and similarly for $\mathbf{E}_{-1}$) satisfies Eqs. (S55)–(S56) but not Eq. (S28), so it is no longer an isolated resonance $\{\psi_n\}$ of the active cavity; it is a mixture of resonances. One can quantify the amount of mixing by projecting $\mathbf{E}_1$ onto the two dominant resonances,

$$\frac{\mathbf{E}_1}{\sqrt{\langle \varepsilon_c \mathbf{E}_1^2 \rangle}} \approx \sum_{n=1}^{2} \frac{\langle \varepsilon_c \mathbf{E}_1 \cdot \psi_n \rangle}{\sqrt{\langle \varepsilon_c \mathbf{E}_1^2 \rangle}\sqrt{\langle \varepsilon_c \psi_n^2 \rangle}} \frac{\psi_n}{\sqrt{\langle \varepsilon_c \psi_n^2 \rangle}}, \tag{S57}$$

where $\mathbf{E}_1^2 = \mathbf{E}_1 \cdot \mathbf{E}_1$ and $\psi_n^2 = \psi_n \cdot \psi_n$. For the near-EP laser in the main text at $D_2^{\rm th}$, the normalized projection factors are

$$\left| \frac{\langle \varepsilon_c \mathbf{E}_1 \cdot \psi_1 \rangle}{\sqrt{\langle \varepsilon_c \mathbf{E}_1^2 \rangle}\sqrt{\langle \varepsilon_c \psi_1^2 \rangle}} \right|^2 = 0.26, \quad \left| \frac{\langle \varepsilon_c \mathbf{E}_1 \cdot \psi_2 \rangle}{\sqrt{\langle \varepsilon_c \mathbf{E}_1^2 \rangle}\sqrt{\langle \varepsilon_c \psi_2^2 \rangle}} \right|^2 = 0.80, \tag{S58}$$

which confirms that $\mathbf{E}_1$ has significant contributions from both resonances.

An intermediate case is possible for Hermitian degeneracies arising from symmetry,[6,7] where ζ can be small because there is no Petermann factor enhancement, but the ratio $\gamma_\parallel/\omega_{\rm d}$ can be large. Since ζ is small, $\mathbf{E}_{-1}$ is negligible, and Eqs. (S55)–(S56) approximately decouple. The decoupled eigenproblem Eq. (S55) for $\mathbf{E}_1$ differs from Eq. (S28) because of the nonzero χ_+ (which is proportional to $\gamma_\parallel$) induced by a dynamic inversion in the perturbation. Here, the stability eigenmode $\mathbf{E}_1$ can also be a mixture of the near-degenerate active-cavity resonances[6,7] but is monochromatic.

5. PALT FOR DIRECT-BANDGAP SEMICONDUCTOR LASERS

In the preceding sections, we considered the Maxwell–Bloch equations for an ensemble of two-level atoms, which captures the essential properties of the nonlinear gain in a minimal form. Here, we consider a more quantitative model for a semiconductor gain medium.[10–12] **Supplementary Fig. 1** shows the band structure of a direct-bandgap semiconductor near the bandgap. The electrons comply with the density matrix equation, Eq. (S2), with the same Hamiltonian expressed by Eq. (S1). But unlike the two-level system, here the electron's Hilbert space has more than two dimensions. Let $\{|B,\mathbf{k}\rangle\}$ be the eigenstates of $\hat{H}_0$, where B represents valence ($B = v$) or conduction ($B = c$) band and $\mathbf{k}$ the Bloch wave vector. Let N be the total density of electrons on both bands. $N_{Bk} = N\langle B,\mathbf{k}|\hat{\rho}|B,\mathbf{k}\rangle$ is the electron population at band B at $\mathbf{k}$. The light emission/absorption occurs when electrons transit between the valence band and the conduction band. In direct-bandgap semiconductor lasers, these radiative inter-band transitions only occur at the same $\mathbf{k}$, shown by the black arrows in **Supplementary Fig. 1**. It means $\langle B_2,\mathbf{k}_2|\hat{\mathbf{r}}|B_1,\mathbf{k}_1\rangle = \mathbf{0}$ when $\mathbf{k}_1 \neq \mathbf{k}_2$. We also assume spatial symmetry, $\langle B,\mathbf{k}|\hat{\mathbf{r}}|B,\mathbf{k}\rangle = \mathbf{0}$ for $B = v, c$. Upon that, we can derive the (observable) optical polarization $\tilde{\mathbf{P}}$ as

$$\tilde{\mathbf{P}} = -eN\mathrm{tr}(\hat{\rho}\hat{\mathbf{r}}) = \sum_{\mathbf{k}} (\mathbf{P}_{\mathbf{k}} + \mathbf{P}_{\mathbf{k}}^*), \tag{S59}$$

where

$$\mathbf{P}_{\mathbf{k}} = N\langle c,\mathbf{k}|\hat{\rho}|v,\mathbf{k}\rangle \mathbf{R}_{\mathbf{k}}^*, \tag{S60}$$

$$\mathbf{R}_{\mathbf{k}} = -e\langle c,\mathbf{k}|\hat{\mathbf{r}}|v,\mathbf{k}\rangle. \tag{S61}$$

Similar to the derivation of Eqs. (S7)–(S9), we decompose Eq. (S2) under $\{|B, \mathbf{k}\rangle\}$ to get the equations for $N_{v\mathbf{k}}$, $N_{c\mathbf{k}}$, and $\mathbf{P_k}$,

$$\frac{\partial}{\partial t} N_{v\mathbf{k}} = \sum_{B',\mathbf{k}'} \gamma_{(B'\mathbf{k}')(v\mathbf{k})} N_{B'\mathbf{k}'} - \sum_{B',\mathbf{k}'} \gamma_{(v\mathbf{k})(B'\mathbf{k}')} N_{v\mathbf{k}} - \frac{1}{i\hbar} \tilde{\mathbf{E}} \cdot (\mathbf{P_k} - \mathbf{P_k}^*), \tag{S62}$$

$$\frac{\partial}{\partial t} N_{c\mathbf{k}} = \sum_{B',\mathbf{k}'} \gamma_{(B'\mathbf{k}')(v\mathbf{k})} N_{B'\mathbf{k}'} - \sum_{B'\mathbf{k}'} \gamma_{(v\mathbf{k})(B'\mathbf{k}')} N_{c\mathbf{k}} + \frac{1}{i\hbar} \tilde{\mathbf{E}} \cdot (\mathbf{P_k} - \mathbf{P_k}^*), \tag{S63}$$

$$\frac{\partial}{\partial t} \mathbf{P_k} = -(i\omega_\mathbf{k} + \gamma_{\perp \mathbf{k}}) \mathbf{P_k} + \frac{1}{i\hbar} (\mathbf{E} \cdot \mathbf{R_k})(N_{c\mathbf{k}} - N_{v\mathbf{k}}) \mathbf{R}^*_\mathbf{k}, \tag{S64}$$

At each $\mathbf{k}$ point, $\omega_\mathbf{k} = \omega_v(\mathbf{k}) - \omega_c(\mathbf{k})$, where $\hbar\omega_v(\mathbf{k})$ and $\hbar\omega_c(\mathbf{k})$ are the energies of valence and conduction band, respectively. $\gamma_{\perp \mathbf{k}}$ is the dephasing rate. The summation terms in Eq. (S62) and Eq. (S63) describe incoherent transitions, including spontaneous emission, pumping, and intra-band non-radiative scattering caused by atomic collisions. It is neither feasible nor necessary to determine each $\gamma_{(B'\mathbf{k}')(B\mathbf{k})}$. The intra-band scattering happens much faster than inter-band transitions. Therefore, in the time scale of our interest, the electron occupation of each band is at a quasi-equilibrium, allowing us to simplify Eq. (S62) and Eq. (S63) by statistics,

$$\frac{\partial}{\partial t} N_{v\mathbf{k}} = -\gamma_{v\mathbf{k}} [N_{v\mathbf{k}} - N f_v(\mathbf{k})] - \frac{1}{i\hbar} \tilde{\mathbf{E}} \cdot (\mathbf{P_k} - \mathbf{P_k}^*), \tag{S65}$$

$$\frac{\partial}{\partial t} N_{c\mathbf{k}} = -\gamma_{c\mathbf{k}} [N_{c\mathbf{k}} - N f_c(\mathbf{k})] + \frac{1}{i\hbar} \tilde{\mathbf{E}} \cdot (\mathbf{P_k} - \mathbf{P_k}^*), \tag{S66}$$

where $\gamma_{v\mathbf{k}}$ and $\gamma_{c\mathbf{k}}$ are relaxation rates, and $f_v(\mathbf{k})$ and $f_c(\mathbf{k})$ are Fermi–Dirac distributions,

$$f_{v,c}(\mathbf{k}) = \frac{1}{e^{(\hbar\omega_{v,c}(\mathbf{k}) - \mu_{v,c})/(k_B T)} + 1}. \tag{S67}$$

$\mu_{v,c}$ is the chemical potential of valence/conduction band. k_B is the Boltzmann constant, and T is temperature in Kelvin. At thermal equilibrium, $\mu_c = \mu_v$, so $f_v(\mathbf{k}) > f_c(\mathbf{k})$. When being pumped, $\mu_c > \mu_v$. So, the pumping strength can be defined as $\mu_\Delta = \mu_c - \mu_v$. To turn on the laser, μ_Δ must be large enough to achieve population inversion, $f_v(\mathbf{k}) < f_c(\mathbf{k})$.

Eqs. (S64)–(S66) generalize Eqs. (S9)–(S10) to a direct-bandgap semiconductor, which forms the MB equations together with Maxwell's equations, Eq. (S17).

For frequency combs, we consider the trial solution,

$$\mathbf{E}(\mathbf{r}, t) = e^{-i\omega_0 t} \sum_{m=-\infty}^{+\infty} \mathbf{E}_m(\mathbf{r}) e^{-im\omega_\mathrm{d} t}, \tag{S68}$$

$$\mathbf{P_k}(\mathbf{r}, t) = e^{-i\omega_0 t} \sum_{m=-\infty}^{+\infty} \mathbf{R_k}^* P_{\mathbf{k}m}(\mathbf{r}) e^{-im\omega_\mathrm{d} t}, \tag{S69}$$

$$N_{B\mathbf{k}}(\mathbf{r}, t) = \sum_{m=-\infty}^{+\infty} N_{B\mathbf{k}m}(\mathbf{r}) e^{-im\omega_\mathrm{d} t}. \; B = c, v. \tag{S70}$$

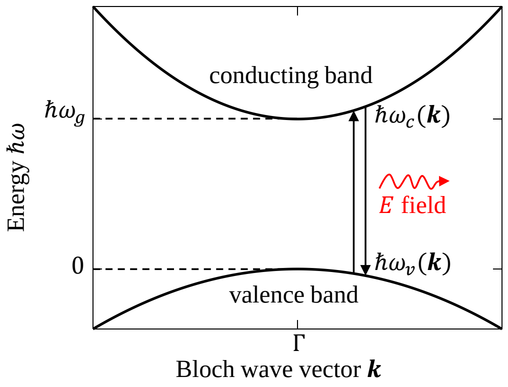

Supplementary Fig. 1. The band structure of a direct-bandgap semiconductor.

Applying the same mathematical steps that lead to Eqs. (18-20) in the Method section of the main text, we can show that Eqs. (S68)–(S70) provide an exact solution of the MB equations for a direct-bandgap semiconductor under rotating-wave approximation. Therefore, we conclude that the same EP comb phenomenon also exists here.

The above derivations lead to

$$\bar{N}_{v\mathbf{k}} = Nf_v(\mathbf{k})\bar{\delta} - \bar{\bar{\Gamma}}_{v\mathbf{k}}(\bar{\bar{E}}^{\dagger}_{\mathbf{k}}\bar{P}_{\mathbf{k}} - \bar{\bar{E}}_{\mathbf{k}}\bar{P}^{*}_{\mathbf{k}-}), \tag{S71}$$

$$\bar{N}_{c\mathbf{k}} = Nf_c(\mathbf{k})\bar{\delta} + \bar{\bar{\Gamma}}_{c\mathbf{k}}(\bar{\bar{E}}^{\dagger}_{\mathbf{k}}\bar{P}_{\mathbf{k}} - \bar{\bar{E}}_{\mathbf{k}}\bar{P}^{*}_{\mathbf{k}-}), \tag{S72}$$

$$\bar{P}_{\mathbf{k}} = \bar{\bar{\Gamma}}_{\mathbf{k}+}\bar{\bar{E}}_{\mathbf{k}}\bar{D}_{\mathbf{k}}, \tag{S73}$$

$$\bar{P}^{*}_{\mathbf{k}-} = \bar{\bar{\Gamma}}^{\dagger}_{\mathbf{k}-}\bar{\bar{E}}^{\dagger}_{\mathbf{k}}\bar{D}_{\mathbf{k}}. \tag{S74}$$

The notation follows that of the main text:

Column vectors: $(\bar{P}_{\mathbf{k}})_m = P_{\mathbf{k}m}$, $(\bar{P}^{*}_{\mathbf{k}-})_m = P^{*}_{\mathbf{k},-m}$, $(\bar{N}_{B\mathbf{k}})_m = N_{B\mathbf{k}m}$ for $(B = c, v)$, $\bar{D}_{\mathbf{k}} = \bar{N}_{c\mathbf{k}} - \bar{N}_{v\mathbf{k}}$ and $(\bar{\delta})_m = \delta_m$.

Matrices: $(\bar{\bar{E}})_{mn} = \mathbf{E}_{m-n}\cdot\mathbf{R}_{\mathbf{k}}$, $(\bar{\bar{\Gamma}}_{B\mathbf{k}})_{mn} = \delta_{m-n}/[\hbar(m\omega_{\rm d}+i\gamma_{B\mathbf{k}})]$, $(\bar{\bar{\Gamma}}_{\mathbf{k}\pm})_{mn} = \delta_{m-n}/[\hbar(\pm m\omega_d+\omega_0-\omega_{\mathbf{k}}+i\gamma_{\perp\mathbf{k}})]$. Then we substitute Eqs. (S73)–(S74) into Eqs. (S72)–(S71) to solve for $\bar{D}_{\mathbf{k}}$,

$$\bar{D}_{\mathbf{k}} = N[\bar{\bar{I}} - (\bar{\bar{\Gamma}}_{v\mathbf{k}} + \bar{\bar{\Gamma}}_{c\mathbf{k}})(\bar{\bar{E}}^{\dagger}_{\mathbf{k}}\bar{\bar{\Gamma}}_{\mathbf{k}+}\bar{\bar{E}}_{\mathbf{k}} - \bar{\bar{E}}_{\mathbf{k}}\bar{\bar{\Gamma}}^{\dagger}_{\mathbf{k}-}\bar{\bar{E}}^{\dagger}_{\mathbf{k}})]^{-1}[f_c(\mathbf{k}) - f_v(\mathbf{k})]\bar{\delta}, \tag{S75}$$

which is analogous to Eq. (12) in the main text. Substituting the polarization in Eqs. (S73), (S69), (S59) into Maxwell's equations Eq. (S17), we get the wave equation

$$-\nabla\times\nabla\times\mathbf{E}_m + \frac{\omega_m^2}{c^2}\left(\varepsilon_c + \frac{i\sigma}{\omega_m\varepsilon_0}\right)\mathbf{E}_m = -\frac{\omega_m^2}{c^2}\sum_{\mathbf{k}}(\bar{\bar{\Gamma}}_{\mathbf{k}+}\bar{\bar{E}}_{\mathbf{k}}\bar{D}_{\mathbf{k}})_m\mathbf{R}_{\mathbf{k}}{}^{*}, \tag{S76}$$

which is analogous to Eq. (11) in the main text. Note that although Eqs. (S75)–(S76) are derived for semiconductor lasers, they also apply to multi-level gain media.

6. SYSTEM PARAMETERS AND PROPERTIES

Supplementary Fig. 2a shows the parameters of the one-dimensional (1D) EP laser considered in the main text. The pumping profile is

$$D_{\rm p}(x) = \begin{cases} 0.5D_{\max}\left[1-\cos\left(\frac{2\pi x}{L}\right)\right], & 0 < x < L \\ 0, & \text{elsewhere} \end{cases} \tag{S77}$$

with $L = 2050$ nm being the length of the first cavity. The two cavities' lengths, the geometric parameters of the middle distributed Bragg reflector (DBR), and the conductivity σ are tuned coarsely (in 10 nm steps) to bring the system close to an EP around ω_{ba} at the first lasing threshold $D_1^{\rm th}$. The absorption in the passive cavity is fixed at $\sigma/\epsilon_0 = 4.9$ ps^{-1} when $D_{\max}$ is below the comb threshold $D_2^{\rm th} = D_{\rm c}^{\rm th} = 0.064$ and raised linearly with $D_{\max}$ above the comb threshold.

Supplementary Fig. 3 compares (1) the comb spectrum in Fig. **3g** of the main text at $\Delta = 0$, $D_{\max} = 0.2$, $\sigma/\varepsilon_0 = 8.1$ ps^{-1} with (2) the nonlinearity-frozen active-cavity eigenvalues $\{\tilde{\omega}_n\}$ of Eq. (S28) for the same parameters and using the lasing mode $\mathbf{E}_0$ from SALT (which predicts the system to stay single-mode at $D_{\max} = 0.2$). We see that the two center comb lines $\{\omega_0, \omega_1\}$ lie close to the two near-degenerate active-cavity eigenvalues, $\{{\rm Re}(\tilde{\omega}_0), {\rm Re}(\tilde{\omega}_1)\}$. The other comb lines do not line up with any additional cavity modes (which are far away in frequency) since they are generated by the nonlinear gain through dynamic four-wave mixing rather than by the other cavity modes. Note that ${\rm Im}(\tilde{\omega}_1)$ has moved down on the complex-frequency plane (*i.e.*, becomes more lossy) compared to its value at the comb threshold $D_{\max} = 0.064$ (see Fig. **2d** of the main text) because of the increased absorption σ.

7. PALT SOLUTION THROUGH VOLUME INTEGRAL EQUATION

For the 1D system above, $\theta = \hat{z}, \mathbf{E}_m = E_m\theta^{*}$, $\partial_y = \partial_z = 0$, and Eq. (15) from the main text can be simplified as

$$\frac{d^2}{dx^2}E_m + \frac{\omega_m^2}{c^2}\left(\varepsilon_c + \frac{i\sigma}{\omega_m\varepsilon_0}\right)E_m = -\frac{\omega_m^2}{c^2}P_m. \tag{S78}$$

Here, the gain-induced polarization $P_m = P_m(E_0, E_{\pm 1}, ...)$ on the right-hand side depends nonlinearly on the fields at all frequencies $\{\omega_m\}$ and is given by Eq. (14) and Eq. (12). We want to solve this system of nonlinear equations for the complex-valued $\{E_m(x)\}_m$ and the real-valued ω_0 and $\omega_{\rm d}$.

In prior work on SALT, the analogous equations are numerically solved in differential form by expanding $E_m(x)$ in a set of threshold constant flux (TCF) modes[1] or with finite-difference frequency-domain (FDFD) discretization.[2] Both approaches would be suitable for a typical system away from EP. However, the hyper sensitivity near an EP amplifies numerical error and requires an unusually high accuracy, and we found both methods to be inefficient for the present system given the desired accuracy.

Here, we adopt a volume-integral approach instead. We define the retarded Green's function $G_m(x, x')$ of the cold cavity at frequency ω_m by

$$\frac{\partial^2}{\partial x^2} G_m(x, x') + \frac{\omega_m^2}{c^2}\left[\varepsilon_c(x) + \frac{i\sigma(x)}{\omega_m \varepsilon_0}\right] G_m(x, x') = -\frac{\omega_m^2}{c^2}\delta(x - x'), \tag{S79}$$

with an outgoing boundary condition. Given $G_m(x, x')$, Eq. (S78) can be written in integral form as

$$E_m(x) = \int_{-\infty}^{\infty} G_m(x, x') P_m(x') dx'. \tag{S80}$$

Since the cold cavity in Eq. (S79) consists of a 1D stack of piecewise-constant permittivity values, we can efficiently solve for the Green's function $G_m(x, x')$ semi-analytically with a high precision; such solution captures all linear effects of the cold cavity, including the DBR confinement, the oscillatory nature of the resonances, and their radiation loss. Then, Eq. (S80) only needs to handle effects of the nonlinear gain, which changes relatively slowly and smoothly. Also, since the gain-induced polarization P_m vanishes outside of the pumped region, the integration range in Eq. (S80) can be reduced to the active cavity $0 \leq x' \leq L$.

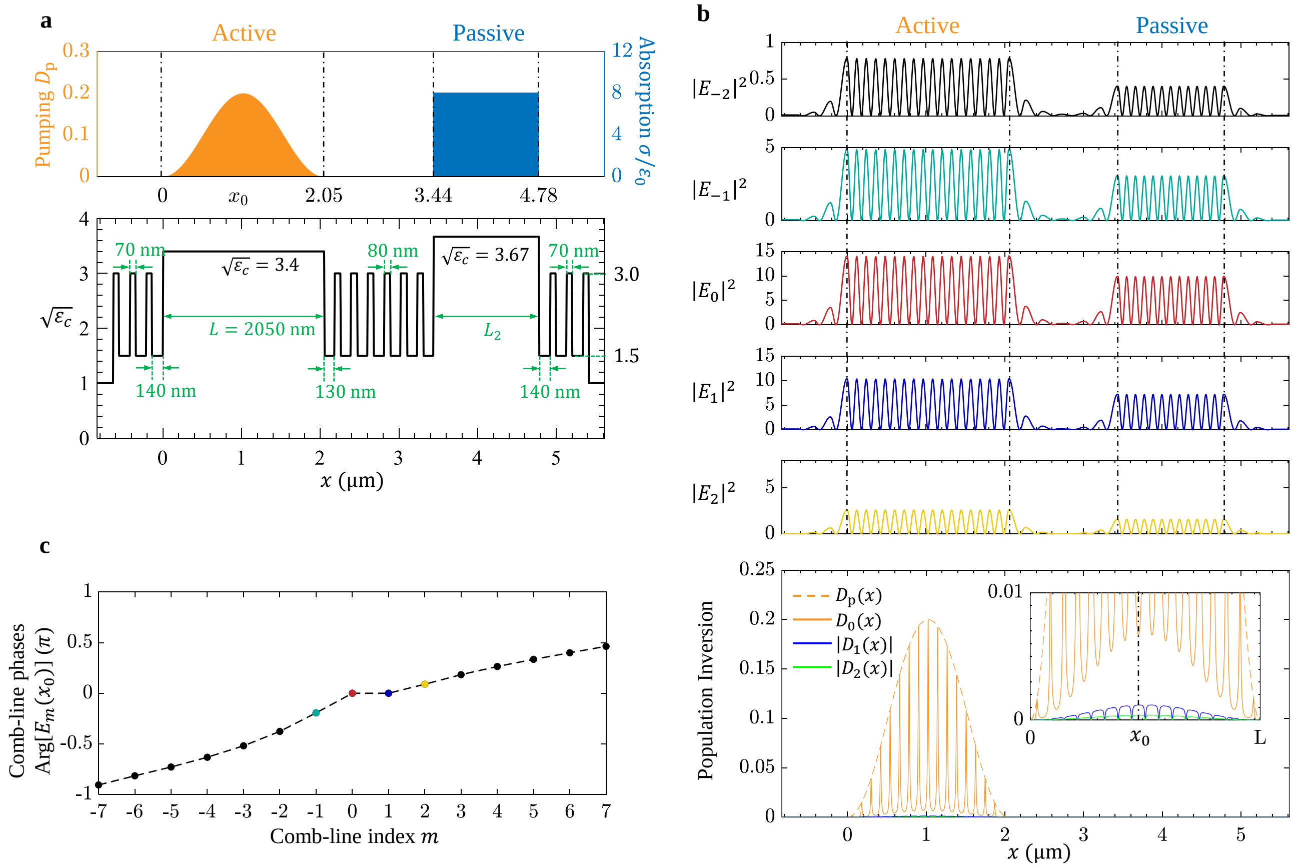

Supplementary Fig. 2. **a** Parameters of the EP laser considered in the main text. **b** Spatial profiles of the intensity and of the inversion at different frequency components, for the EP comb in Fig. 3a of the main text (with $D_{\max} = 0.2$, $\sigma/\epsilon_0 = 8.1$ ps^{-1} and $L_2 = 1340$ nm). **c** The phases of the electrical fields $E_m(x_0)$ with $-7 \leq m \leq 7$.

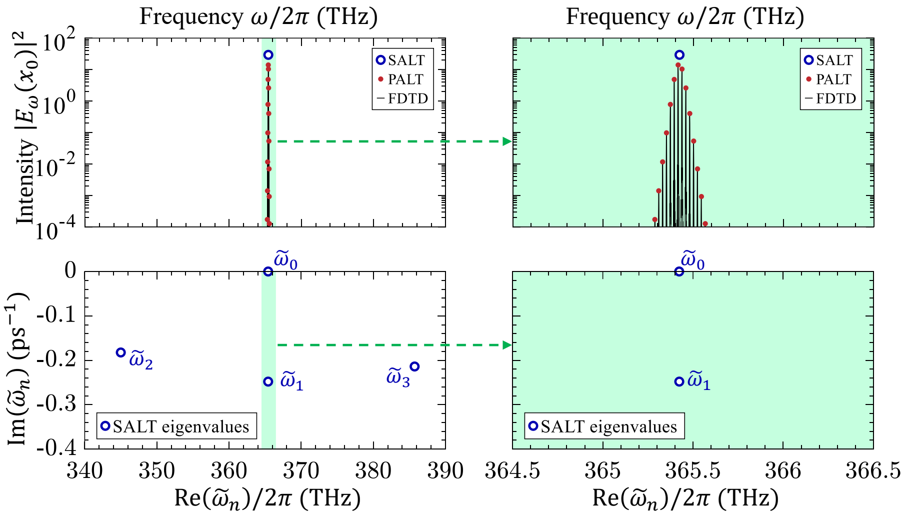

Supplementary Fig. 3. Comparison between the EP comb spectrum (upper panels) and the resonant frequencies of the active cavity from SALT through Eq. (S28) (lower panels). $\tilde{\omega}_0$ and $\tilde{\omega}_1$ form the near-EP pair. The next nearest resonant modes, $\tilde{\omega}_2$ and $\tilde{\omega}_3$, are separated from $\tilde{\omega}_{0,1}$ by the free spectral range of the active cavity. The right panels show zoom-ins near the EP frequency.

For numerical calculation, we truncate the frequency summation to $2M+1$ frequencies with $m = -M, -M+1, \cdots, M$ and approximate the integration of Eq. (S80) with the trapezoidal rule

$$E_m(x_j) = \sum_{k=0}^{K} G_m(x_j, x_k) P_m(x_k) \Delta x_k, \tag{S81}$$

where $x_k = kL/K$, $\Delta x_0 = \Delta x_K = L/(2K)$, and $\Delta x_k = L/K$ for $k = 1, \cdots, K-1$. Evaluating Eq. (S81) with $j = 0, 1, \cdots, K$ yields $(2M+1)(K+1)$ complex-valued equations with $(2M+1)(K+1)$ complex-valued scalar unknowns: $\{E_m(x_j)\}$ with $m \in [-M, M]$ and $j \in [0, K]$. Additionally, there are two more real-valued unknowns: ω_0 and ω_{d}. To match the number of equations and the number of unknowns, we recognize that when $\{E_m(x_j)\}$ is a solution, $\{e^{-i\Psi_0 - im\Psi_{\text{d}}} E_m(x_j)$ is also a solution for any real-valued Ψ_0 and Ψ_{d}. So, we choose Ψ_0 and Ψ_{d} such that $E_0(x_0)$ and $E_1(x_0)$ are real-valued. Then, we have the following $2(2M+1)(K+1)$ real-valued variables

$$\text{Variables} = \begin{bmatrix} \text{Im}[E_{-M}(x_0)] & \text{Re}[E_{-M}(x_0)] & \dots & \text{Re}[E_{-M}(x_K)] \\ \vdots & \vdots & \vdots & \vdots \\ \omega_0 & \text{Re}[E_0(x_0)] & \dots & \text{Re}[E_0(x_K)] \\ \omega_{\text{d}} & \text{Re}[E_1(x_0)] & \dots & \text{Re}[E_1(x_K)] \\ \vdots & \vdots & \vdots & \vdots \\ \text{Im}[E_M(x_0)] & \text{Re}[E_M(x_0)] & \dots & \text{Re}[E_M(x_K)] \end{bmatrix}, \tag{S82}$$

with the same number of real-valued equations. We solve this system of nonlinear equations with the `fsolve` function in MATLAB. Given $E_m(x_j)$ at these $K+1$ points, we can evaluate Eq. (S81) at other values of x_j to obtain a continuous profile $E_m(x)$ anywhere (including with x outside the pumped cavity or outside the DBR mirrors). For the example in this paper, we use $M = 7$ and $K = 200$, which is sufficient to reach four digits of accuracy for ω_{d}. In contrast, a differential finite-difference approach will require over 100 times more spatial grid points to reach a comparable accuracy.

We adopt the same volume integral approach to solve for ω_0 and E_0 in the single-mode lasing regime of Eq. (S23) and to solve the stability eigenvalue problem of Eqs. (S55)–(S56).

8. FDTD SIMULATIONS OF THE MAXWELL–BLOCH EQUATIONS

To validate the PALT predictions, we additionally carry out direct integration of the MB equations using the finite-difference time-domain (FDTD) method. Here, we directly work with the real-valued fields, $\mathbf{E}$, $\mathbf{B}$, $\text{Re}(\mathbf{P})$, $\text{Im}(\mathbf{P})$ and

D in Eqs. (S11)–(S14) without introducing the rotating-wave approximation. Details on the FDTD discretization are given in Ref..[13] For the 1D simulation here, we implement the outgoing boundary analytically.[14]

Due to the enhanced sensitivity of EPs, a very fine spatial discretization is required for the FDTD simulations to reach enough accuracy to reproduce the PALT results for the EP laser described in Sec. 6 above. Here, we set the spatial grid size to be $\Delta x = 0.25$ nm and the time step size to be $\Delta t = \Delta x/c$. This requires over one billion time steps to evolve the system by just one relaxation time $1/\gamma_{\parallel} = 1$ ns of the gain medium. Further reducing Δx can make FDTD agree even closer to PALT but will incur higher computing costs.

In the absence of noise, the laser spectrum at long times should have zero linewidths. The linewidths in Fig. 2(g,h) of the main text come from a finite temporal window in the FDTD simulations. A frequency comb solution features electrical field

$$\tilde{\mathbf{E}}(\mathbf{r},t) = \mathbf{E}(\mathbf{r},t) + \mathbf{E}^*(\mathbf{r},t) = e^{-i\omega_0 t}\sum_m \mathbf{E}_m(\mathbf{r})e^{-im\omega_d t} + \text{c.c.}, \quad -\infty < t < \infty. \tag{S83}$$

Fourier transform (FT) of the infinite time series in Eq. (S83) yields

$$\text{FT}\Big\{\tilde{\mathbf{E}}(\mathbf{r},t)\Big\} = 2\pi\sum_m \mathbf{E}_m(\mathbf{r})\delta(\omega-\omega_m) + 2\pi\sum_m \mathbf{E}^*_m(\mathbf{r})\delta(\omega+\omega_m), \tag{S84}$$

which has zero linewidths. In practice, even after the transient behaviors settle away in a FDTD simulation, we will only have access to data within a finite duration: one section of Eq. (S83) in time. Let T be the duration of the long-time data taken from $\tilde{\mathbf{E}}(\mathbf{r},t)$. The truncated data is then expressed as $w(t)\tilde{\mathbf{E}}(\mathbf{r},t)$, where $w(t)$ is a finite-sized window function confined within $-0.5T < t < 0.5T$. Let $W(\Delta\omega)$ be the Fourier transform of $w(t)$. Then, the Fourier transform of the practical (truncated) data is

$$\text{FT}\Big\{w(t)\,\tilde{\mathbf{E}}(\mathbf{r},t)\Big\} = \sum_m \mathbf{E}_m(\mathbf{r})W(\omega-\omega_m) + \sum_m \mathbf{E}^*_m(\mathbf{r})W(\omega+\omega_m). \tag{S85}$$

The raw FDTD data correspond to a rectangular window for $w(t)$, whose spectrum $W(\Delta\omega)$ is a sinc function with bandwidth $2\pi/T$, which has side peaks that decay slowly as $(\Delta\omega T)^{-1}$. The side peaks may corrupt the underlying comb spectrum. A brute-force way to suppress the side peaks is to use a sample duration T that is many orders of magnitude beyond the temporal periodicity $\tau = 2\pi/\omega_{\text{d}}$, so that the side peaks are negligible at $\Delta\omega = m\omega_{\text{d}}$. But doing so is wasteful and computationally costly since $\tau \approx 50$ ps is large in the near-EP system studied here. To more efficiently suppress the side peaks of the window function spectrum, we use a Hann window instead,

$$w(t) = \begin{cases} \frac{1}{2}\left[1+\cos\left(\frac{2\pi t}{T}\right)\right], & -0.5T < t < 0.5T, \\ 0, & \text{otherwise.} \end{cases} \tag{S86}$$

Its Fourier transform $W(\Delta\omega)$ has a wider center lobe, but the side peaks decay much faster as $(\Delta\omega T)^{-3}$, allowing a much smaller sampling duration T. Here, $W(\Delta\omega = 0) = T/2$, so in Fig. 2(g,h) of the main text we plot

$$\mathbf{E}_\omega(\mathbf{r}) \equiv \frac{2}{T}\text{FT}\Big\{w(t)\,\tilde{\mathbf{E}}_{\text{FDTD}}(\mathbf{r},t)\Big\}, \tag{S87}$$

such that the peaks of $|\mathbf{E}_\omega|^2$ will equal $|\mathbf{E}_m|^2$ in the long-time limit.

After $\tilde{E}(x,t)$ and $D(x,t)$ settle down, we continue to run the simulation for $T = 800$ ps $\approx 16\tau$ while recording $\tilde{E}(x_0,t)$ at $x = x_0$. We record one data point per $20\Delta t$, which is over a hundred data points per oscillation, well above the Nyquist sampling rate. A fast Fourier transform (FFT)[15] is then used to compute the spectrum. To ensure that the frequency grid of FFT does not accidentally miss the narrow peak of each comb line, we pad zeros on both sides of the temporal data after the Hann window and before the FFT.

9. EXACT-EP LASER

As shown in **Supplementary Fig. 3**, we did not tune to an exact EP for the system considered in the main text. There, the system is tuned close to an EP at the first threshold ($D_{\max} = D_1^{\text{th}}$) and stays close to the EP in the single-mode lasing regime when gain saturation is accounted for. The near-EP single-mode lasing state remains stable until when the pumping strength reaches the comb threshold $D_2^{\text{th}} = D_{\text{c}}^{\text{th}}$.

In this section, we consider the less realistic but conceptually interesting question: what happens when we tune the system to an exact EP? An EP below the lasing threshold D_1^{th} (such as the EP in **Supplementary Fig. 3**) is not accessible at steady state. So, we are interested in an EP above D_1^{th}.

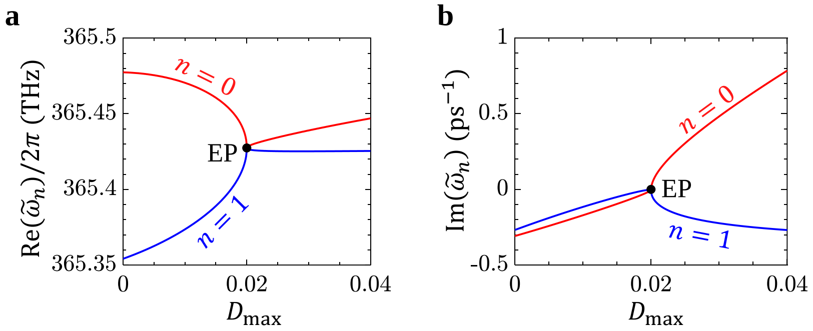

Supplementary Fig. 4. Evolution of the two eigenvalues $\tilde{\omega}_0$ and $\tilde{\omega}_1$ of Eq. (S28) as a function of the pumping strength $D_{\max}$. **a** The real part of $\tilde{\omega}_0$ (red) and $\tilde{\omega}_1$ (blue). **b** The imaginary part of $\tilde{\omega}_0$ (red) and $\tilde{\omega}_1$ (blue). The exceptional point is marked as a black dot in both **a** and **b**. The system is the same as Supplementary Fig. **2**a except that the passive cavity length is tuned to 1339.985 nm and the material loss is tuned to $\sigma/\varepsilon_0 = 4.021$ ps^{-1} to bring the system to an exact lasing EP at $D_{\max} = D_{\rm EP} = 0.02000$. The $\mathbf{E}_0(\mathbf{r})$ in Eq. (S21) is fixed to the $\mathbf{E}_0(\mathbf{r})$ at $D_{\max} = D_{\rm EP}$ to make the operator $\hat{O}$ linear throughout the evolution.

To be clear, here we consider an (almost) exact EP of the active-cavity wave operator $\hat{O}$ in Eq. (S28). For a given pump strength, after solving the nonlinear Eq. (S27) to obtain the single-mode lasing state $\mathbf{E}_0(\mathbf{r})$, we consider the saturated gain $D_0(\mathbf{r})$ with the $E_0(\mathbf{r})$ in Eq. (S21) fixed, which defines the linear operator $\hat{O}$ in Eq. (S28). We then tune the system parameters with higher precisions to bring this nonlinearity-frozen operator $\hat{O}$ to an EP. By tuning the length of the passive cavity to 1339.985 nm and the absorption to $\sigma/\varepsilon_0 = 4.021$ ps^{-1}, we bring the system very close to a single-mode lasing EP at $D_{\max} = D_{\rm EP} = 0.02000 > D_1^{\rm th} = 0.01336$. Here, the eigenvalue difference $|\tilde{\omega}_0 - \tilde{\omega}_1| \approx 0.02$ ps^{-1} is around 3000 times smaller than the free spectral range of the overall cavity. The Petermann factors are $K_1 = 3704$ and $K_2 = 369$, much larger than those of the near-EP system in Sec. 6. **Supplementary Fig. 4** shows the evolution of the two eigenvalues $\tilde{\omega}_0$ and $\tilde{\omega}_1$ of Eq. (S28) as a function of $D_{\max}$, where we make the operator $\hat{O}$ linear throughout the evolution by fixing the $E_0(\mathbf{r})$ in Eq. (S21) to the $E_0(\mathbf{r})$ at the EP with pumping strength $D_{\max} = D_{\rm EP}$.

Like the near-EP example considered in the main text, if one were to apply SALT to this exact-EP system, one would incorrectly conclude that the system stays in the single-mode lasing regime over a wide range of pumping values with $D_{\max} > D_1^{\rm th}$, including at $D_{\max} = D_{\rm EP}$, for the same reason as described in Sec. 6. However, when applying the PALT single-mode stability analysis by solving Eqs. (S55)–(S56) on the EP state, we find a stability eigenvalue of $\omega_d = (0.0557 + 0.0254i)$ ps^{-1} with a positive imaginary part, indicating that this exact-EP state is in fact unstable. This result is consistent with Ref. [9], which also found a single-mode lasing EP to be unstable in a different coupled cavity system but did not find what state the system would evolve into.

Equipped with PALT, we can now find out what state the laser evolves to when the single-mode lasing EP is unstable. We fix the other system parameters and gradually increase the pumping strength, starting from zero pump. With the PALT stability analysis, we find the single-mode solution to be stable only within a very small range of pumping strengths, $D_1^{\rm th} = 0.01336 < D_{\max} < D_2^{\rm th} = D_{\rm c}^{\rm th} = 0.01346$. At the comb threshold $D_{\rm c}^{\rm th}$, the laser develops multiple frequencies with a repetition rate of 2.3 GHz. **Supplementary Fig. 5** shows the spectra at different $D_{\max}$, comparing the exact PALT and the SALT predictions. Because the absorption is not raised together with the pumping strength, the comb spacing increases above the comb threshold. At $D_{\max} = D_{\rm EP} = 0.02000$, the laser operates as a stable comb with 14.5 GHz repetition rate. In other words, the exact lasing EP is unreachable. The system evolves into an EP comb before reaching the unstable exact EP.

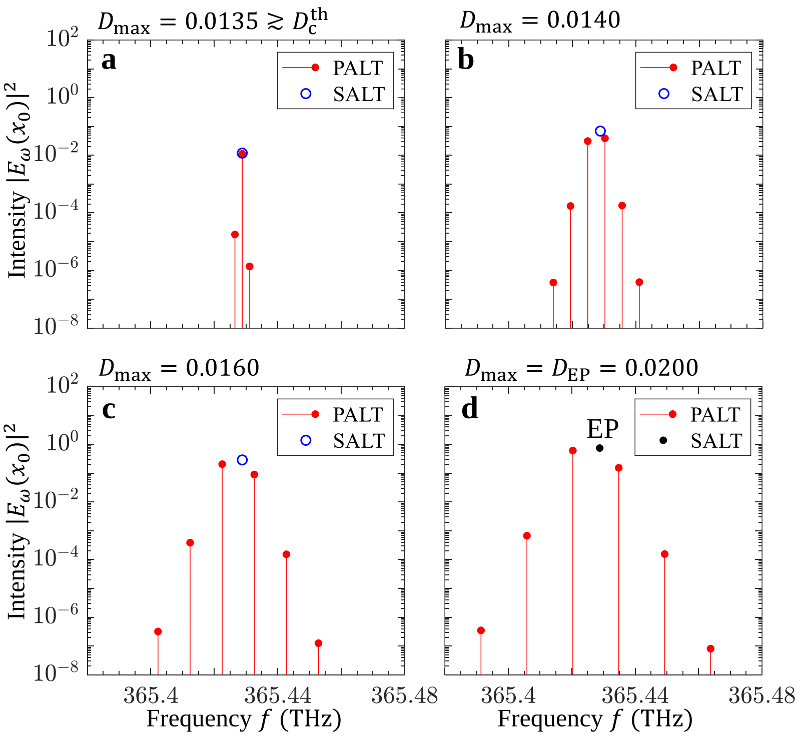

Supplementary Fig. 5. The spectrum of the exact-EP laser in Supplementary Fig. **4** at different pumping strengths, calculated by PALT and SALT. **a** The spectrum at a D_{max} of 0.0135, which is slightly above the comb threshold. **b**–**c** The evolution of the spectrum as D_{max} increases from the comb threshold towards the exact exceptional point. **d** The spectrum at the exact exceptional point, $D_{max} = D_{EP}$, where the laser operates as a stable comb even though SALT incorrectly D_{max} increases from predicts a single-mode EP behavior. $x_0 \approx 1.0$ μm is the location marked in Fig. 2b of the main text.

[1] Ge, L., Chong, Y. D. & Stone, A. D. Steady-state *ab initio* laser theory: Generalizations and analytic results. *Phys. Rev. A* **82**, 063824 (2010).
[2] Esterhazy, S. *et al.* Scalable numerical approach for the steady-state *ab initio* laser theory. *Phys. Rev. A* **90**, 023816 (2014).
[3] Sauvan, C., Wu, T., Zarouf, R., Muljarov, E. A. & Lalanne, P. Normalization, orthogonality, and completeness of quasinormal modes of open systems: the case of electromagnetism [invited]. *Opt. Express* **30**, 6846–6885 (2022).
[4] Balanis, C. A. *Advanced engineering electromagnetics, Appendix II* (John Wiley & Sons, 2012).
[5] Pick, A. *et al.* General theory of spontaneous emission near exceptional points. *Opt. Express* **25**, 12325–12348 (2017).
[6] Burkhardt, S., Liertzer, M., Krimer, D. O. & Rotter, S. Steady-state *ab initio* laser theory for fully or nearly degenerate cavity modes. *Phys. Rev. A* **92**, 013847 (2015).
[7] Liu, D. *et al.* Symmetry, stability, and computation of degenerate lasing modes. *Phys. Rev. A* **95**, 023835 (2017).
[8] Benzaouia, M., Cerjan, A. & Johnson, S. G. Is single-mode lasing possible in an infinite periodic system? *Appl. Phys. Lett.* **117**, 051102 (2020).
[9] Benzaouia, M., Stone, A. D. & Johnson, S. G. Nonlinear exceptional-point lasing with *ab initio* Maxwell–Bloch theory. *APL Photonics* **7**, 121303 (2022).
[10] Cerjan, A., Chong, Y., Ge, L. & Stone, A. D. Steady-state *ab initio* laser theory for N-level lasers. *Opt. Express* **20**, 474–488 (2012).
[11] Chow, W. W. & Koch, S. W. *Semiconductor-laser fundamentals: physics of the gain materials, Chapter 2* (Springer Science & Business Media, 2013).
[12] Cerjan, A., Chong, Y. D. & Stone, A. D. Steady-state *ab initio* laser theory for complex gain media. *Opt. Express* **23**, 6455–6477 (2015).
[13] Cerjan, A., Pick, A., Chong, Y. D., Johnson, S. G. & Stone, A. D. Quantitative test of general theories of the intrinsic laser linewidth. *Opt. Express* **23**, 28316–28340 (2015).
[14] Mur, G. Absorbing boundary conditions for the finite-difference approximation of the time-domain electromagnetic-field equations. *IEEE Trans. Electromagn. Compat.* **EMC-23**, 377–382 (1981).
[15] Frigo, M. & Johnson, S. The design and implementation of FFTW3. *Proc. IEEE* **93**, 216–231 (2005).
